# Antipathogen genes and the replacement of disease-vectoring mosquito populations: a model-based evaluation

**DOI:** 10.1111/eva.12219

**Published:** 2014-10-10

**Authors:** Michael A Robert, Kenichi W Okamoto, Fred Gould, Alun L Lloyd

**Affiliations:** 1 Department of Mathematics and Biomathematics Graduate Program, North Carolina State UniversityRaleigh, NC, USA; 2 Department of Biology and Department of Mathematics and Statistics, University of New MexicoAlbuquerque, NM, USA; 3 Department of Entomology, North Carolina State UniversityRaleigh, NC, USA; 4 Fogarty International Center, National Institutes of HealthBethesda, MD, USA

**Keywords:** *Aedes aegypti*, antipathogen genes, dengue fever, female-killing, ordinary differential equation model, reduce and replace

## Abstract

Recently, genetic strategies aimed at controlling populations of disease-vectoring mosquitoes have received considerable attention as alternatives to traditional measures. Theoretical studies have shown that female-killing (FK), antipathogen (AP), and reduce and replace (R&R) strategies can each decrease the number competent vectors. In this study, we utilize a mathematical model to evaluate impacts on competent *Aedes aegypti* populations of FK, AP, and R&R releases as well as hybrid strategies that result from combinations of these three approaches. We show that while the ordering of efficacy of these strategies depends upon population life history parameters, sex ratio of releases, and switch time in combination strategies, AP-only and R&R/AP releases typically lead to the greatest long-term reduction in competent vectors. R&R-only releases are often less effective at long-term reduction of competent vectors than AP-only releases or R&R/AP releases. Furthermore, the reduction in competent vectors caused by AP-only releases is easier to maintain than that caused by FK-only or R&R-only releases even when the AP gene confers a fitness cost. We discuss the roles that density dependence and inclusion of females play in the order of efficacy of the strategies. We anticipate that our results will provide added impetus to continue developing AP strategies.

## Introduction

Because many insect-vectored diseases remain endemic despite implementation of traditional control measures, several novel genetic pest management (GPM) vector control strategies have been proposed to reduce vector capacity and hence transmission of pathogens (Whitten and Foster 1975; Hemingway et al. 2006; Sinkins and Gould 2006). These strategies have generally aimed to achieve either vector population reduction (Whitten 1969; Heinrich and Scott 2000; Thomas et al. 2000; Gong et al. 2005; Alphey et al. 2010) or replacement (Davis et al. 2001; Burt 2003; Hay et al. 2010) via field release of engineered strains of the vector. Both population reduction and population replacement strategies have been explored theoretically (Foster et al. 1988; Schliekelman and Gould 2000; Davis et al. 2001; Magori and Gould 2006; Atkinson et al. 2007; Huang et al. 2007, 2009; Phuc et al. 2007; Deredec et al. 2008; Yakob et al. 2008; White et al. 2010; Marshall and Hay 2011; Ward et al. 2011), and several engineered mosquito strains have been developed (Catteruccia et al. 2005; Fu et al. 2010; Mathur et al. 2010; Labbé et al. 2012). Most of these engineered strains are designed with the intention of releasing males of the strain as females are a biting nuisance and, in the absence of genes conferring disease refractoriness, are capable of transmitting disease pathogens; however, recent releases of females infected with *Wolbachia* show promise that females could be included in releases if these released females were incapable of transmitting pathogens (Hoffmann et al. 2011).

Among the GPM approaches that have seen tangible progress are female-killing (FK) strategies (Heinrich and Scott 2000; Thomas et al. 2000). In an FK strategy, a transgene is inserted into the vector's genome that causes conditional female-specific lethality, but does not affect males. To enable the rearing of the transgenic strain, the female-specific lethal element is designed to be conditionally expressed. For example, when certain genetically engineered FK strains of the mosquito species that transmit both dengue fever and chikungunya viruses, *Aedes aegypti* and *Aedes albopictus,* are reared on a diet containing tetracycline, the lethal effects of the FK gene are repressed; tetracycline is not found in the pest's diet in the wild, so offspring of the released individuals are subject to the effects of the FK gene (Fu et al. 2010; Labbé et al. 2012). When FK males are released and mate with wild-type females, female offspring that inherit a single copy of the FK gene do not survive. Males inheriting the FK gene can survive and pass the gene to their offspring, which is predicted to make FK strategies more efficient than traditional sterile insect technique (SIT) strategies in which neither male nor female offspring survive (Schliekelman and Gould 2000; Phuc et al. 2007; Black et al. 2011). At least one FK construct has been engineered into mosquito species (Fu et al. 2010; Labbé et al. 2012), and laboratory cage tests have shown that repeated releases of FK mosquitoes into a caged wild-type population of *Ae. aegypti* can lead to elimination of the wild-type population (Wise de Valdez et al. 2011). Field cage tests of this same construct only resulted in some reduction in population density, suggesting a high fitness cost to the specific engineered strain in a tropical environment (Facchinelli et al. 2013).

In a previous study, we proposed and theoretically evaluated a reduce and replace (R&R) strategy aimed at short-term reduction and long-term replacement of a vector by releasing individuals homozygous for an FK gene as well as an antipathogen (AP) gene rendering the vector incapable of transmitting disease-causing pathogens (Robert et al. 2013). We explored several potential R&R release scenarios with a relatively simple deterministic mathematical model and showed that R&R releases were more effective at reducing the number of competent vectors (i.e., adult females capable of transmitting disease) than comparable FK releases. In a follow-up study, we explored R&R releases with a stochastic, spatially explicit model simulating a neighborhood in the city of Iquitos, Peru (Okamoto et al. 2013). Although the R&R strategy was still predicted to be more effective than the FK strategy, we found that under many conditions sustaining a high level of replacement of the native population with a population incapable of transmitting disease via R&R releases was unlikely. This was in part due to the influences of genetic drift when population size was low and to spatial heterogeneity as the population recovered from reduction, neither of which effects is included in the deterministic model. Taken together, these two studies introduced the R&R approach and provided insight into how R&R can impact the population and genetic structure of one specific mosquito species, *Ae. aegypti*. These differences between the outcomes of the relatively simple deterministic model and the detailed, stochastic model emphasize the utility of assessing potential vector control strategies with a variety of models before development and implementation of these strategies.

Although R&R may be more effective at reducing competent vector populations than FK releases alone under comparable release scenarios, the lack of AP allele fixation observed in the detailed, stochastic model of *Ae. aegypti* raises the need to further evaluate R&R against related strategies that exploit the strengths of R&R, FK, and AP strategies. FK-only strategies have been well studied theoretically (Schliekelman and Gould 2000; Gould and Schliekelman 2004; Atkinson et al. 2007; Phuc et al. 2007), while AP-only strategies (at least in the absence of gene drive) have only been theoretically examined in a single article that does not compare it with other approaches (Rasgon 2009) as well as another recent follow-up study by the authors of this paper that compared the FK-only, R&R, and AP-only strategies in a detailed stochastic, spatial model (Okamoto et al. 2014). In addition to R&R, FK-only, or AP-only strategies, control programmes could be developed that combine these three approaches. For instance, a programme that releases FK individuals prior to beginning releases of AP or R&R individuals would reduce the population before attempting to replace the population with incompetent vectors (Carvalho et al. 2013). Similarly, R&R releases prior to AP-only releases would allow for population replacement to begin while reduction occurs, but could be timed so that AP releases begin before population density is too low [perhaps in an attempt to avoid the effects of low population size observed in Okamoto et al. (2013)]. While FK, AP, and R&R releases have been explored independently in previous models (Foster et al. 1988; Schliekelman and Gould 2000; Rasgon 2009; Robert et al. 2013), to our knowledge, programmes involving combinations of releases of FK, AP, and R&R strains have not yet been systematically compared using a modeling framework.

In this paper, we develop a model based on the general biology of the primary dengue vector *Ae. aegypti* to compare R&R releases with FK-only AP-only, and combination releases. As in Robert et al. (2013), we are interested in a population that is difficult to drive to extinction with population reduction measures, so we consider a population that is regulated by strong density dependence. We explore the effects of different release strategies on vector population dynamics to elucidate how varying the release ratio, release duration, and the sex of released individuals leads to different magnitudes of reduction in competent vectors. We also explore the influence of fitness cost associated with the AP gene and density dependence on the ability of releases to reduce competent vector density. We study the effects of different release scenarios on population dynamics as releases are occurring (i.e., the transient dynamics) as well as the long-term impact on the competent vector population once releases conducted for a finite period of time come to an end (which could occur if a control programme ends due to logistic reasons).

## Materials and methods

We utilize the ordinary differential equation model described in Robert et al. (2013) which we briefly reintroduce here in the context of our present study. An analogous stochastic model is described in the Supplementary Material.

Our model tracks genes at two different loci in an *Ae. aegypti* population. We assume that the FK and AP genes are located at two independently segregating loci, and we denote the FK and AP alleles as ‘K’ and ‘A’, respectively; the corresponding wild-type alleles at each locus are denoted ‘k’ and ‘a’. This leads to nine possible genotypes (see Table 1).

**Table 1:** Possible genotypes resulting from R&R releases with corresponding fitness values (*w*_i_) and female viability coefficients (*γ*_i_)

*i*	Genotype	*w*_*i*_	*γ*_*i*_
1	KKAA	(1−*c*_*A*_)(1−*c*_*K*_)	0*
2	KkAA	(1−*c*_*A*_)(1−0.5*c*_*K*_)	0
3	kkAA	(1−*c*_A_)	1
4	KKAa	(1−0.5*c*_*A*_)(1−*c*_*K*_)	0
5	KkAa	(1−0.5*c*_*A*_)(1−0.5*c*_*K*_)	0
6	kkAa	(1−0.5*c*_*A*_)	1
7	KKaa	(1−*c*_*K*_)	0*
8	Kkaa	(1−0.5*c*_*K*_)	0
9	kkaa	1	1

* Conditional lethality allows for the release of these females as adults.

Let *J*_*i*_(*t*), *F*_*i*_(*t*), and *M*_*i*_(*t*) be the density of juveniles (larvae and pupae; egg production is modeled implicitly), adult females, and adult males, respectively, of genotype *i* at time *t*. We assume random mating between adults and Mendelian inheritance. The rate *B*_*i*_ (*t*) at which females produce viable larvae of genotype *i* is


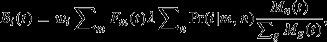
(1)

where *λ* is the per capita rate at which females produce larvae, Pr(*i*|*m*,*n*) is the probability that an offspring of genotype *i* arises from a mating between an adult female of genotype *m* and an adult male of genotype *n* (assuming Mendelian inheritance), and *w*_*i*_ is the fitness of an offspring of genotype *i* relative to that of wild-type offspring. We assume that fitness costs affect the fraction of eggs that survive to the larval stage, and that fitness costs are additive at a single locus and multiplicative across loci. We denote the fitness cost associated with homozygous FK individuals as *c*_K_ and that associated with homozygous AP individuals as *c*_A_. We note that our model can readily accommodate alternative assumptions regarding types of fitness costs (e.g*.,* dominant or recessive) as well as the life stages on which fitness costs act (e.g*.,* mating or adult viability).

Let *μ*_*J*,_ *μ*_*F*,_ and *μ*_*M*_ denote the per capita density-independent mortality rates of juveniles, adult females, and adult males, respectively. Juveniles experience additional density-dependent mortality at per capita rate (*αJ*)^*β*−1^, where *J* is the total juvenile density and *β* and *α* are parameters governing the strength of density dependence and, together with other parameters, they determine the equilibrium population density (Bellows 1981). The strength of density dependence governs how quickly the population returns toward equilibrium following perturbations. Strong density dependence (e.g., higher values of *β*) accelerates this return. Juveniles emerge as mature adults at per capita rate *ν*, and we assume that adults emerge with a 1:1 sex ratio in the absence of FK effects. The rate of emergence of female adults is scaled by a viability coefficient, *γ*_*i*_, where *γ*_*i*_ = 1 for viable genotypes and *γ*_*i*_ = 0 otherwise (see Table 1).

We assume transgenic releases occur continuously. Let 
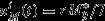
 and 
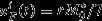
 be the rates at which adult males and adult females, respectively, of genotype *i* are released. Here, *r* is the initial weekly release ratio of transgenic individuals to the equilibrium wild-type male population density 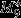 (the factor 7 scales the weekly release rate to daily rate). Note that the rate at which individuals are released is taken to remain constant even when population densities decline. Release rates that vary as the population declines could be considered as has been done in a previous model (e.g., Atkinson et al. 2007), although this is not a common practice in large-scale SIT programmes. In this paper, we consider the releases of three distinct genotypes: homozygous FK (KKaa, *i* = 7), homozygous AP (kkAA, *i* = 3), and homozygous R&R (KKAA, *i* = 1). Note that conditional lethality allows for the release of adult females carrying FK genes.

Because we track nine genotypes of juveniles, adult females, and adult males, the model description above leads to the following system of 27 ordinary differential equations, where *i* represents the genotype of each class; model parameters, together with their default values, are in Table 2.


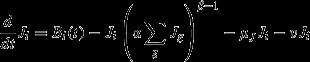



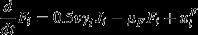



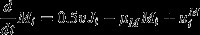
(2)

**Table 2:** Description of model parameters with default values and references for default values

Parameter	Description	Default value	References
*μ*_*J*_	Density-independent juvenile mortality rate (per capita)	0.03 day^−1^	Rueda et al. (1990)
*μ*_*M*_	Adult male mortality rate (per capita)	0.28 day^−1^	Muir and Kay (1998), Fouque et al. (2006)
*μ*_*F*_	Adult female mortality rate (per capita)	0.10 day^−1^	Muir and Kay (1998), Fouque et al. (2006)
*λ*	Average rate of larval production by females (per capita)	8 day^−1^	Harrington et al. (2001), Styer et al. (2007)
*ν*	Rate of emergence to adulthood (per capita)	0.14 day^−1^	Muir and Kay (1998)
*α*	Density dependence parameter	2 × 10^−4^ juveniles^*β*−1^ · day^1/(*β*−1)^	–
*β*	Strength of density dependence	3.4	–
*c*_A_	Fitness cost associated with antipathogen allele	0	–
*c*_K_	Fitness cost associated with female-killing allele	0	–
*w*_*i*_	Fitness of genotype *i*	See Table 1	–
*γ*_*i*_	Female viability coefficient of genotype *i*	See Table 1	–
*r*	Weekly release ratio of transgenic individuals to wild-type males	1	–
*T*	Duration of release	100 days	–
*T*_*s*_	Time at which combination releases switch	50 days	–

We consider six different strategies that arise from combinations of FK, AP, and R&R releases. Along with releases that include only one approach (R&R-only, AP-only, and FK-only), we consider three strategies (FK/AP, R&R/AP, and FK/R&R) that switch from one approach to another at a switching time, *T*_s_. In the FK/AP strategy, FK releases are conducted first, followed by a period of AP-only releases. In the R&R/AP strategy, R&R releases are conducted and followed by AP-only releases. In the FK/R&R strategy, FK-only releases are conducted before R&R releases. We compare these combined strategies against single-approach releases lasting the same total duration.

We assess the efficacy of all release strategies by observing changes in the competent vector population that result from releasing transgenic mosquitoes into an entirely wild-type population at demographic equilibrium. We compare the strategies by considering the order of efficacy from most to least effective at reducing competent vector density. For each strategy, we consider male-only, bi-sex (50% male, 50% female), and female-only releases and compare releases of each type that lead to the same total number of mosquitoes being released. Although releases of R&R and AP females would be possible because these females cannot transmit pathogens, releasing FK-only females is undesirable because they are competent vectors. For this reason, in scenarios that would involve the release of FK females, we replace FK females with FK males for our analyses, even when comparing strategies that include female-only and bi-sex releases of the R&R and AP-only strains. For example, in a ‘female-only’ FK/AP release, FK males are released followed by AP females.

## Results

Here we compare results for a number of scenarios in which each of the different release strategies is implemented. In each figure, we present the density of competent vectors relative to the wild-type equilibrium density. For all analyses, the values for the release ratio, release duration, and switch time are held at the default values in Table 2 unless noted otherwise. In the supplementary material, we discuss how these and other parameters influence our results (Figures S8, S13, S14). For all results presented here, corresponding results obtained from the stochastic model are presented in the supplementary material.

### Release switch time for male-only releases

**Figure 1:**
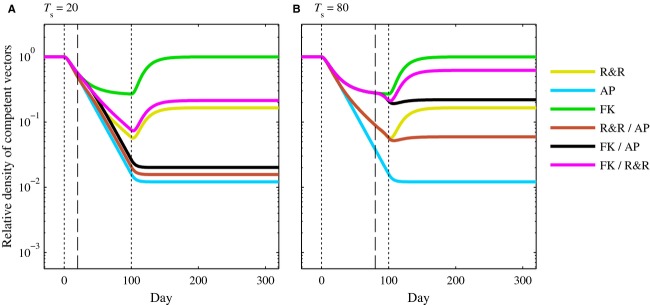
Relative density of competent vectors when male-only releases were conducted at a 1:1 release ratio (*r* = 1) for *T* = 100 days. Vertical black dotted lines represent the time at which releases began (*t* = 0) and ended (*t* = 100). For combination strategies, the vertical black dashed line represents the time at which a switch was made between approaches. Here, the time of switch was *T*_s_ = 20 (A) and *T*_s_ = 80 (B). All other parameter values are the default values listed in Table 2. Note the vertical axis is on a log scale.

We simulated 100-day male-only releases at a 1:1 release ratio (total engineered males:total wild-type males per week). For the combined approaches, we switched from the first to second strain after 20 (Fig. 1A) or 80 (Fig. 1B) days. Regardless of the switch time, the AP-only strategy always led to the greatest reduction in competent vectors during and after releases, followed by the R&R/AP combination (note that the vertical axis in all figures is on a log scale). The FK/AP combination led to greater reduction in competent vectors than R&R-only for the earlier switch time (Fig. 1A), but for the later switch time, R&R-only reduced the competent vector density more than the FK/AP strategy (Fig. 1B). We note that the differences between the long-term density of competent vectors following AP-only, R&R/AP, and FK/AP releases were small when the switch time was earlier. Indeed, in the stochastic model, fluctuations due to demographic stochasticity were larger than the differences seen here (Figure S1).

The FK-only strategy always had the smallest impact of the six strategies on competent vectors during and after releases. Note that for the combination of release ratio and strength of density dependence considered here, FK releases were only capable of reducing the population to a lower equilibrium value and would not drive the population toward extinction. (In Figure S7 of the supplementary material, we compare releases at higher release ratios.) The FK/R&R combination strategy led to greater reduction than FK-only releases but was not as effective as any of the other strategies. These impacts of the FK-only and FK/R&R strategies relative to other strategies remained consistent for all release scenarios we describe throughout this paper, so henceforth, we omit these results to simplify the presentation and discussion of results.

### Male-only, female-only, and bi-sex releases

**Figure 2:**
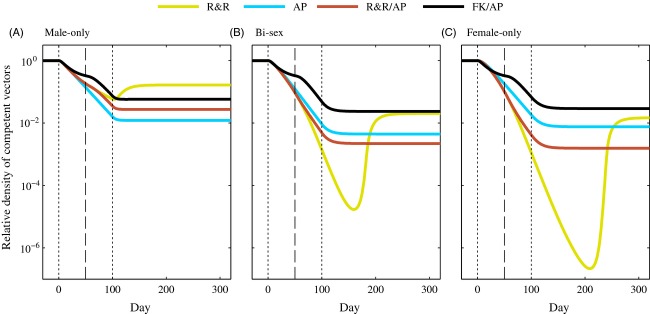
Relative density of competent vectors when releases were conducted at a 1:1 release ratio (*r* = 1) for *T* = 100 days with male-only (A), bi-sex (B), and female-only (C) releases. Vertical black dotted lines represent the time at which releases began (*t* = 0) and ended (*t* = 100), and the vertical black dashed line represents the time at which the switch between approaches in combination strategies occurred (*T*_s_ = 50). All other parameter values are the default values listed in Table 2. Note the vertical axis is on a log scale.

We compared 100-day male-only, bi-sex, and female-only releases at a 1:1 (total engineered adults:total wild-type males per week) release ratio (Fig. 2). For each of the combination strategies, we considered a switching time of *T*_s_ = 50 days after releases began. Bi-sex and female-only releases of each of the strategies led to greater reduction in competent vectors than comparable male-only releases. When releases included females, R&R releases led to the greatest reduction during the transient period, but R&R/AP releases led to the greatest long-term reduction in competent vectors for both bi-sex and female-only releases, followed by AP-only releases (Fig. 2B,C). We note that the difference in the efficacy for bi-sex AP-only and R&R/AP releases was minor, which is emphasized in results from the stochastic model (Figure S2). Bi-sex AP-only releases caused greater long-term reduction in competent vectors than similar male-only and female-only releases. As with male-only releases, increasing the switch time for bi-sex and female-only releases led to the FK/AP strategy having less impact on the competent vector population density than the R&R-only strategy (Figure S9).

### Density dependence

**Figure 3:**
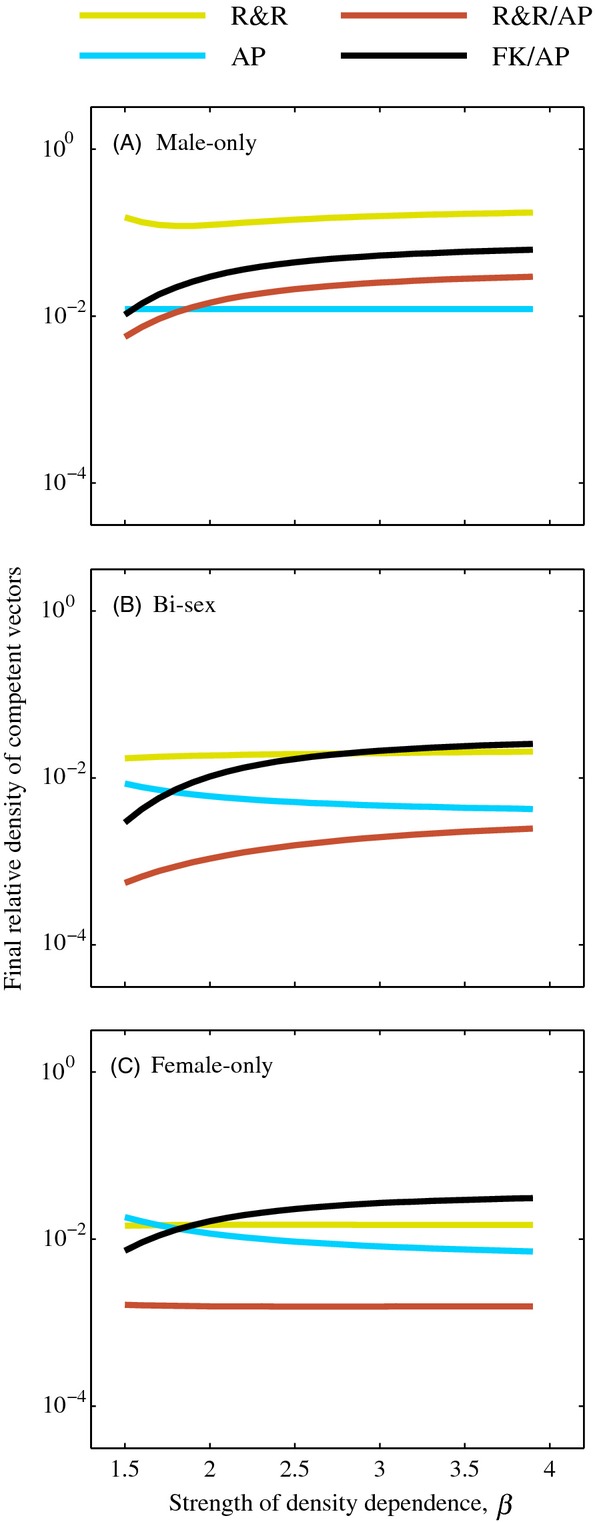
Long-term relative density of competent vectors for populations regulated by different strengths of density dependence when releases were conducted at a 1:1 (*r* = 1) release ratio for *T* = 100 days with male-only (A), bi-sex (B), and female-only (C) releases. For combination strategies, the switch occurred after *T*_*s*_ = 50 days. All other parameter values are the default values listed in Table 2. Note the vertical axis is on a log scale.

Next, we assessed how density dependence affects the ability of each of the strategies to decrease competent vector density. Density-dependent population regulation can either help or hinder vector control strategies and has been of particular concern in the development of transgenic strategies (Barclay 2005; Atkinson et al. 2007; Legros et al. 2009; Walsh et al. 2011). We simulated male-only, bi-sex, and female-only releases of each strategy for 100 days (with *T*_*s*_ = 50 for combination strategies) at a 1:1 release ratio for populations regulated by different strengths of density dependence by varying the value of *β* in equation 2. We observed both the long-term (Fig. 3) and minimum (Figure S11) relative competent vector population density resulting from each release scenario. In general, the strength of density dependence did not affect the ordering of efficacy of the strategies on reducing competent vectors for bi-sex and female-only releases. The exception was that for very weak density dependence, FK/AP releases led to lower long-term density in competent vectors than AP-only releases, while for stronger density dependence, AP-only releases led to lower long-term density of competent vectors than FK/AP releases (Fig. 3B,C). For bi-sex and female-only releases, the R&R strategy led to the greatest reduction during the transient period for most strengths of density dependence considered here (Figure S11B,C).

For male-only releases, the strength of density dependence determined which of the strategies was most effective at reducing the density of competent vectors in the transient period, but had less impact on long-term reduction (Fig. 3A, Figure S11A). For weak density dependence, R&R led to the greatest reduction in competent vector density during the transient period while AP and FK/AP releases led to the least reduction (Figure S11A); R&R/AP releases caused the greatest long-term reduction in competent vectors and R&R-only releases caused the least long-term reduction when density dependence was weak (Fig. 3A). For stronger density dependence, AP releases led to the greatest reduction during the transient period and long term, followed by R&R/AP releases (Fig. 3A, Figure S11A), although the difference in long-term reduction caused by AP and R&R/AP releases was minor (Figure S3A).

### Sex ratio of releases

**Figure 4:**
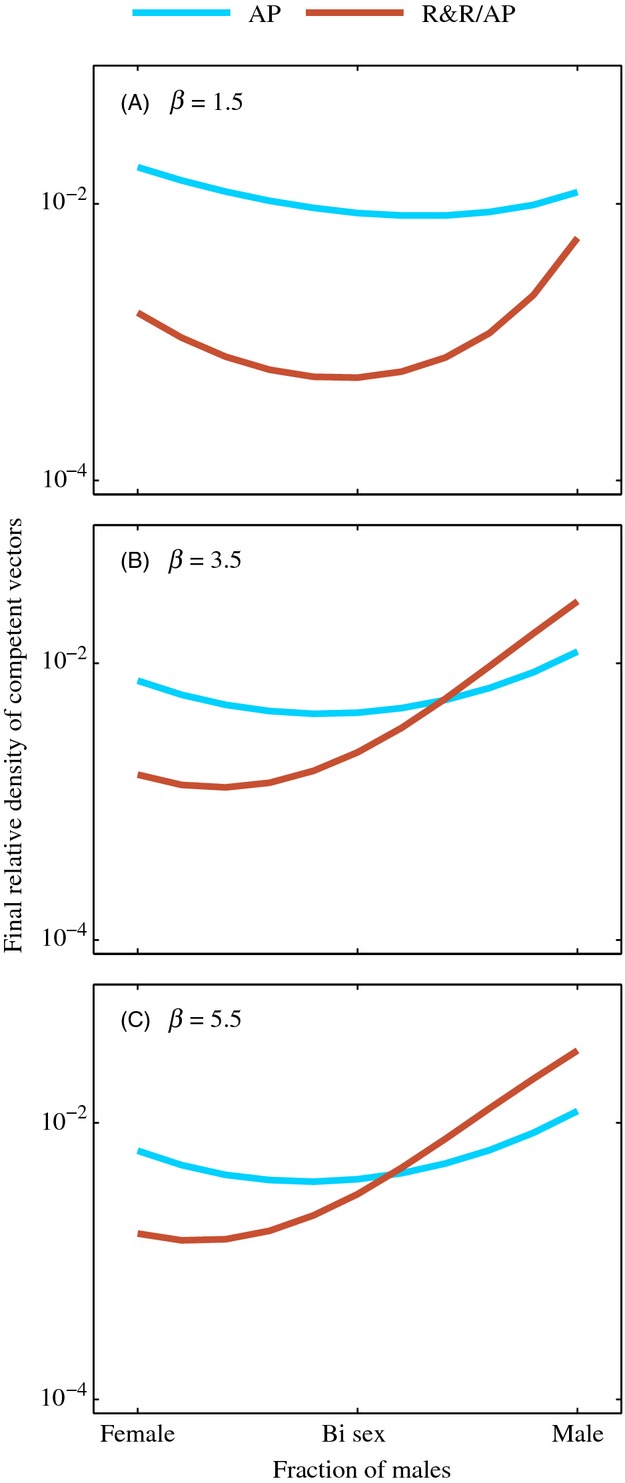
Long-term relative density of competent vectors when the sex ratio and strength of density dependence varied for releases. AP-only and R&R/AP releases are conducted at a 1:1 release ratio (*r* = 1) for *T* = 100 days with a strength of density dependence of *β* = 1.5 (A), *β* = 3.5 (B), and *β* = 5.5 (C). Switch time for R&R/AP releases was *T*_s_ = 50. All other parameter values are the default values listed in Table 2. Note the vertical axis is on a log scale.

Because the sex ratio of released adults had a noticeable impact on the order of efficacy of R&R/AP and AP-only strategies, we revisit the impacts of sex ratio of releases by further comparing these two release strategies across sex ratios that spanned the spectrum from male-only to female-only releases for three different strengths of density dependence (Fig. 4). As before, we compared releases at a 1:1 release ratio for *T* = 100 days with combination strategies switching at *T*_s_ = 50 days. In the supplementary material, we also compared four release strategies across three different strengths of density dependence in the presence and absence of a fitness cost associated with the AP gene (Figure S10). The most effective strategy changed with both the sex ratio and the strength of density dependence. For weak density dependence, R&R/AP releases were more effective at reducing competent vectors than AP-only releases regardless of the sex ratio of releases (Fig. 4A). For higher strengths of density dependence, AP-only releases led to the greatest reduction in competent vectors at higher male-to-female ratios, whereas R&R/AP releases were most effective at lower male-to-female ratios (Fig. 4B,C). Additionally, the optimal sex ratio for each release changed with the strength of density dependence, generally favoring lower male-to-female ratios as density dependence strengthened. In the supplementary material, we show that both density dependence and fitness cost may impact the influence of sex ratio on the order of efficacy of the strategies (Figure S10).

### Fitness cost

**Figure 5:**
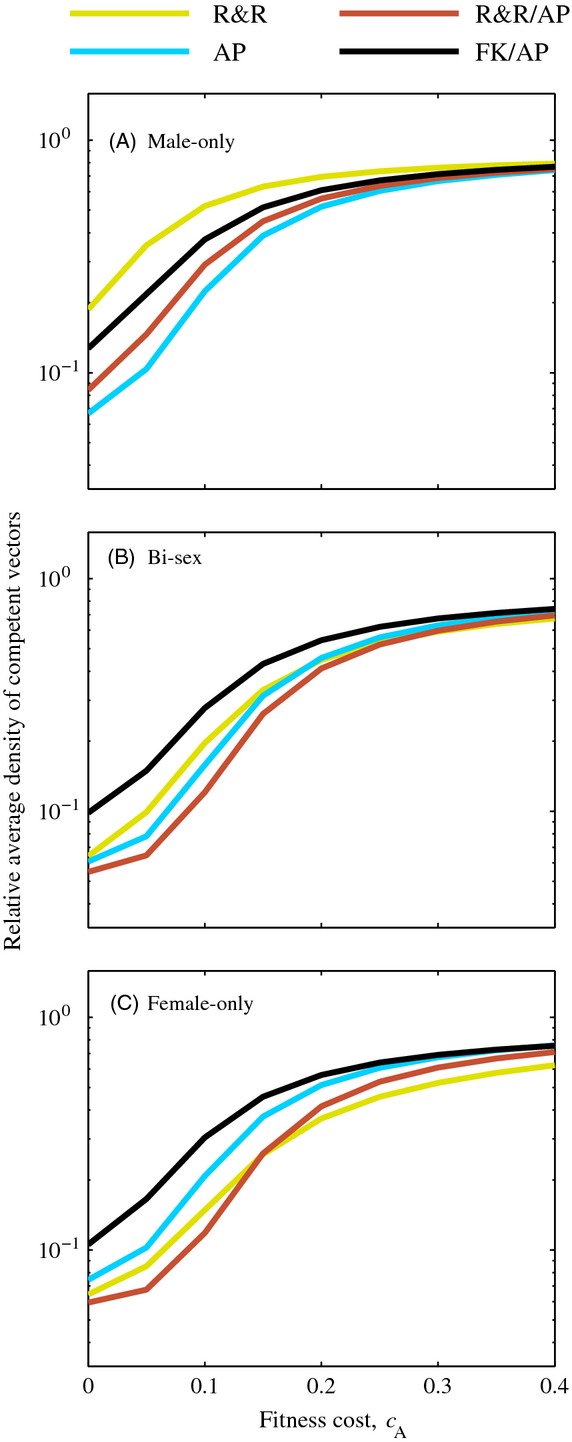
Relative average density of competent vectors between the time releases began (*t*_0_ = 0) and one year after the final day of releases (*t*_f_ = 465) when the antipathogen gene carried a fitness cost. Releases were conducted at a 1:1 (*r* = 1) release ratio for *T* = 100 days with male-only (A), bi-sex (B), and female-only (C) releases. For combination strategies, the switch between approaches occurred after *T*_*s*_ = 50 days. All other parameter values are the default values listed in Table 2. Note the vertical axis is on a log scale.



To explore potential impacts of a fitness cost associated with the AP gene, we compared male-only, bi-sex, and female-only releases for 100 days at a 1:1 release ratio with a switch time of *T*_*s*_ = 50 days under a range of fitness costs associated with the AP gene (Fig. 5, Figure S12). If there is a fitness cost associated with the AP gene, any reduction in the competent vector population will only be temporary (Figure S12), so studying the long-term density here is not particularly informative. To measure the effect on each of the strategies, we calculated the average density of competent vectors over the time period beginning the day releases start (*t*_0_) and ending 1 year after the last day of releases (*t*_f_) relative to the wild-type equilibrium number of females in the absence of control (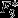). That is, as *F*_9_(*t*) represents the density of wild-type female adults in the population subject to control, the relative average density 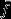 is given by

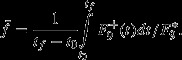
(3)

For the range of fitness costs we studied here, we found no impact on the order of efficacy of the different release types for male-only or bi-sex releases (Fig. 5A). In contrast, female-only releases of the R&R/AP strategy led to lower relative average density of competent vectors than R&R-only releases when the fitness cost was lower than approximately *c* = 0.15, but the opposite was true for higher values of the fitness cost. This difference was minor for all values of the fitness cost considered (Fig. 5C, Figure S5C). For both bi-sex and female-only releases, the FK/AP strategy led to the highest relative average density of these four strategies (Fig. 5B). For female-only releases, the AP-only strategy led to lower relative average density than the FK/AP strategy, but higher relative average density than the R&R and R&R/AP strategies (Fig. 5C).

### Maintenance releases

**Figure 6:**
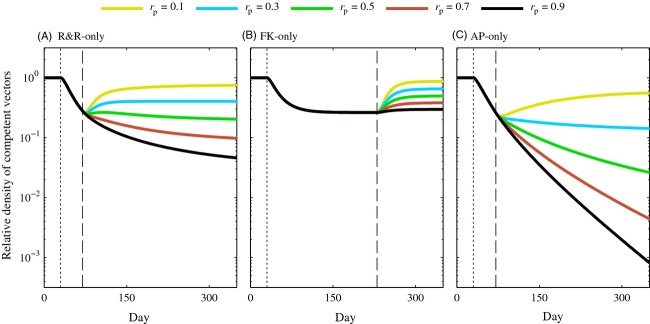
Relative competent vector density in populations where maintenance releases occur when the antipathogen gene has a fitness cost of *c*_*A*_ = 0.2. Male-only R&R (A), female-killing (FK) (B), and anti-pathogen (AP) (C) releases were conducted at a 1:1 (*r* = 1) release ratio until each population under control by the three different strategies reached the same density. The first dotted line represents the beginning of the original releases, and the dashed line represents the end of the original releases and the beginning of maintenance releases. Here, *r*_*p*_ is the fraction of the original releases ratio utilized in maintenance releases. That is, *r*_*p*_ = 1 represents continued releases at the same intensity as the original releases. All other parameter values are the default values listed in Table 2. Note the vertical axis is on a log scale.

If the AP gene has a fitness cost, continuous maintenance releases will be needed to maintain incompetent vectors in a population indefinitely because the fitness cost would result in the AP gene eventually being removed from the population (Figure S12). To compare maintenance releases, we simulated male-only releases of the R&R-only, AP-only, and FK-only strategies at a 1:1 release ratio when the AP gene had an associated fitness cost of *c*_*A*_ = 0.2. In practice, maintenance releases might begin after a fixed duration of releases or once the density of competent vectors falls below some predetermined threshold. We modeled the latter approach by allowing the original releases to occur until competent populations under each release strategy reached the same density, and we considered subsequent maintenance releases by lowering the release ratio to a fraction, *r*_p_, of the original release ratio (Fig. 6). Maintenance of the reduction in competent vectors achieved during the original FK release period required the same ratio as the original releases. Lower fractions of the original release ratio during maintenance releases led to a higher density of competent vectors, although still lower than that of the competent vector density before releases began. ( Fig. 6B). Maintenance releases at a lower fraction of the original release were required to maintain the reduction in competent vectors caused by R&R and AP releases (Fig. 6A,C). Ongoing releases at about 50% of the original release ratio sufficed to prevent a recovery in competent vectors under R&R releases, while releases at about 30% of the original release ratio prevented recovery following AP-only releases. R&R and AP maintenance releases at higher percentages led to further reduction in the density of competent vectors. We note that the fraction of the original release ratio required for maintenance releases will depend on the original release ratio and the magnitude of fitness costs as well as other parameters such as the strength of density dependence.

## Discussion

Most theoretical and empirical assessments of the efficacy of GPM approaches evaluate single genetic mechanisms (Huang et al. 2007). In this paper, we showed that vector control strategies involving strains with FK and AP genes, individually and in combination, can have very different impacts on transient and long-term dynamics of competent vector populations. Indeed, the most effective genetic strategy for control depended upon whether females were included in releases, the time at which combination strategies switched from one approach to the next, the strength of density-dependent mortality, and the magnitude of fitness costs associated with the transgenes. While the results presented here were obtained for a population whose structure and dynamics are relatively simple, they highlight the key role that population dynamics and the design of release strategies play in the success of GPM-based vector control programmes.

In most of the male-only release scenarios that we analyzed, the AP-only releases led to the most effective long-term reduction of competent vectors. This result was unexpected because it has previously been assumed that population reduction, whether prior to or in conjunction with releases of individuals carrying AP genes, would aid the spread of the AP gene (Carvalho et al. 2013; Okamoto et al. 2013; Robert et al. 2013). While simultaneous releases of a strain with FK and AP genes on different chromosomes (as in the R&R releases) led to a rapid reduction in competent vectors, once the population density was low, the two genes were inherited together more frequently, leading to many copies of the AP genes being lost due to the lethal effects of the FK gene (Okamoto et al. 2013; Robert et al. 2013). Although an R&R strain would be designed so that FK and AP genes are not physically linked, linkage disequilibrium at low population densities would still be difficult to avoid. AP-only releases, however, did not suffer from any lethal effects, and AP genes were able to rapidly propagate through the population. Reducing the population prior to AP releases, as in the FK/AP strategy, did not lead to greater reduction in competent vectors than AP-only releases in the scenarios we considered, in part because strong density dependence impeded significant reductions in the vector population density. The period of reduction left less time for AP releases, resulting in a higher long-term density of competent vectors than when AP-only releases were conducted throughout the entire release duration.

AP-only approaches in the absence of gene drive have thus far received little attention as it has been assumed that they would require the release of prohibitively large numbers of transgenic insects. To address this issue, several gene drive systems that cause super-Mendelian inheritance of AP genes have been designed to decrease the number of released transgenic insects required to fix AP genes in a population (Turelli and Hoffmann 1999; Davis et al. 2001; Burt 2003; Chen et al. 2007; Gould et al. 2008; Marshall et al. 2011). In this paper, we showed that despite a fitness cost, the density of competent vectors in a natural population can be reduced more quickly by continuously releasing AP-only strains than by releasing a similar number of FK or R&R mosquitoes, which is consistent with the findings in our previous study comparing FK-only, AP-only, and R&R releases in a more complex model (Okamoto et al. 2014). Moreover, we showed in this paper that the maintenance of competent vector population reduction with AP-only releases requires far fewer mosquitoes than maintenance following FK-only releases.

We found that while AP-only releases typically led to the greatest long-term reduction when only males were released, bi-sex and female-only R&R/AP releases typically caused greater long-term reduction in competent vector density than corresponding AP-only releases. This result is due in part to the interaction between density-dependent mortality and the increase in offspring that resulted from releasing females, which was also observed and discussed in the initial study of the R&R system (Robert et al. 2013). The combination of the increased offspring and the changing density-dependent mortality as the population is reduced and then attempts to rebound toward equilibrium slows the growth rate of the total population and allows for introgression of the AP genes as AP females dominate the mating population of females. This impact of the slower growth rate was also observed when male-only releases were conducted in a population with very weak density dependence (see Figs 3A and 4A), but when density dependence was strong, the total population rebounded too quickly from the R&R phase of male-only R&R/AP releases for the AP phase of the releases to benefit from the initial population reduction (see Figs 2A, 3A, 4A).

Although female-only AP releases led to greater long-term reduction in competent vectors than similar male-only releases, bi-sex AP releases generally led to the greatest reduction due to the impact of competition among released individuals in single-sex releases. For example, in male-only releases, transgenic males had to compete with one another as well as with wild-type males, but when half the number of males was released with an equal number of females, these males could potentially mate with released females as well, reducing competition for mates. Although bi-sex AP releases led to greater reduction in competent vectors than single-sex releases, we found that the proportion of males and females in releases that led to the greatest reduction in competent vectors may fall elsewhere along the spectrum from male-only to female-only releases. In fact, the optimal male-to-female ratio of released adults depended on a number of factors, including the strength of density-dependent mortality and the magnitude of fitness cost associated with the AP gene (Fig. 4, Figure S10). We note, however, that sorting released insects to sex ratios other than male-only, female-only, and bi-sex may be logistically impractical for some insect species, and one should assess the benefit of doing so relative to the effort required to do so before attempting such releases.

Both our deterministic and stochastic models showed that AP-only and R&R/AP releases were most effective at reducing the density of competent vector populations in scenarios where extinction of the population did not occur. For some scenarios, the R&R strategy, particularly in bi-sex and female-only releases, led to elimination of competent vector (e.g., Figure S2B,C); however, we caution an overly optimistic interpretation of these results because on average, R&R releases were not as effective at reducing competent vector density as R&R/AP and AP releases (Figure S2B,C). While total population extinction or at least competent vector elimination would certainly be an ideal scenario, previous models that included more detail showed that such elimination is very unlikely (Legros et al. 2012; Okamoto et al. 2013). The extinction predicted in our study is in fact very optimistic and a product of the limited complexity of the model. In particular, because our model has no spatial heterogeneity, it overestimates the possibility of extinction and competent vector elimination. To truly determine the potential for population extinction, one must consult more detailed models that consider complexity in spatial structure and population dynamics.

In many cases, we found that the strength of density dependence, the mortality of adults (see Figure S13), and the magnitude of fitness cost associated with the AP gene had little effect on the ordering of long-term efficacy of the strategies; however, there were a few scenarios in which the qualitative outcome differed as a result of changing these parameters. For example, one strategy may be more effective than the others in a population in which density dependence is weak while another may be better suited for a population regulated by strong density dependence (e.g., compare Fig. 3, *β* < 2 to *β* > 2). This study reiterates that understanding the role that these parameters play in a control programme is still important, and in particular, life history characteristics of a species should be well understood before choosing a GPM strategy for vector control.

We have shown here that AP-based transgenic strategies hold promise for reducing competent vectors; however, as with any disease control strategy, AP releases are not without potential disadvantages (Alphey et al. 2009; Alphey and Alphey 2014). The relative risk of such control measures should be carefully assessed prior to development and implementation (Benedict et al. 2008; Beech et al. 2009; Hanh et al. 2009). For example, pathogens could evolve to become resistant to antipathogen genes, rendering these transgenes useless against specific strains of a pathogen (Alphey et al. 2009). This potential for the development of resistance is shared with common forms of disease control including the application of insecticides and the distribution of vaccines and antimicrobial medications (Scott et al. 2002; Hill et al. 2005). In addition to the development of resistance, mechanisms designed to protect against transmission of one pathogen could in fact enhance other pathogens (Dodson et al. 2014), which would be particularly concerning for vector species that potentially transmit multiple pathogens. Developers of antipathogen genes should therefore observe the efficacy of the genes closely and consider developing antipathogen genes that are capable of protecting against a wide genetic background of pathogens. In fact, R&R and AP-only strategies could actually be utilized to test the efficacy and evolutionary stability of AP genes that are being considered for gene-drive approaches. The spread of AP genes via R&R and AP-only releases will be more limited to the population into which releases are conducted than gene-drive releases which are designed to spread quickly and are more likely to disseminate beyond the initial population.

Beyond the evolutionary risks associated with AP genes, introducing genetically modified organisms into a population is likely to face major regulatory challenges from governments as well as possible community unease (McNaughton 2012; McNaughton and Duong 2014). To help alleviate this, proper testing in contained environment should be conducted before releases occur in the field (Benedict et al. 2008; Facchinelli et al. 2013; Ramsey et al. 2014). A thorough education of potential risks and benefits should be presented to communities where releases are possible, and public approval should be obtained before such releases occur (Beech et al. 2009; Popovici et al. 2010; McNaughton and Duong 2014). This has previously proved to be effective for *Wolbachia*-based and transgenic population reduction strategies in several instances (Popovici et al. 2010; Amin and Hashim 2014; McNaughton and Duong 2014).

In this study, we evaluated the relative efficacy of several feasible genetic strategies for controlling disease vectors by comparing strategies with a relatively simple, deterministic ordinary differential equation model and its stochastic analog. While more biologically realistic models should be used to further evaluate all of the strategies, our work provides substantial motivation for the empirical assessment of AP, R&R, and combination strategies in the fight against disease vectors. In particular, we stress the need for further theoretical and empirical study of AP approaches, both for AP-only and AP-hybrid strategies, because of their predicted ability to lead to substantial long-term reduction in competent vectors relative to other strategies conducted with a similar degree of effort.

## Supporting Information

**Data S1.** Stochastic model. **Table S1.** Transition rates between compartments of the stochastic model. **Figure S1**. Relative density of competent vectors when male-only releases were conducted at a 1:1 release ratio (*r* = 1) for *T* = 100 days. **Figure S2.** Relative density of competent vectors when releases were conducted at a 1:1 release ratio (*r* = 1) for *T* = 100 days with male-only (A), bi-sex (B), and female-only (C) releases. **Table S2.** Table of percentages of population extinctions observed for R&R releases for different strengths of density dependence, *β*. **Figure S3.** Final relative density of competent vectors for populations regulated by different strengths of density dependence when releases were conducted at a 1:1 (*r* = 1) release ratio for *T* = 100 days with male-only (A), bi-sex (B), and female-only (C) releases. **Figure S4.** Final relative density of competent vectors when the sex ratio varies for releases of transgenic insects when releases were conducted at a 1:1 (*r* = 1) release ratio for *T* = 100 days. **Table S3.** Table of percentages of extinctions observed during R&R releases for different values of fitness cost associated with the AP gene. **Figure S5.** Relative average density of competent vectors between the time releases began (*t*_0_ = 0) and one year after the final day of releases (*t*_*f*_ = 465) when the anti-pathogen gene carries a fitness cost. Releases were conducted at a 1:1 (*r* = 1) release ratio for *T* = 100 days with male-only (A), bi-sex (B), and female-only (C) releases. **Figure S6.** Relative competent vector density in populations where maintenance releases occur when the anti-pathogen gene has a fitness cost. **Figure S7.** The effects of release ratio and switch time on male-only releases. **Figure S8.** Long-term relative competent vector population density resulting from release scenarios that arise from different combinations of release ratio and release duration with male-only (A,D), bi-sex (B,E), and female-only releases (C,F). **Figure S9.** The effect of switch time on female-only releases. Releases were conducted for a total of *T* = 100 days at a 1:1 release ratio (*r* = 1). **Figure S10.** The effects of fitness cost, sex ratio, and density dependence on average relative density of competent vectors. **Figure S11.** Minimum relative density of competent vectors for different strengths of density dependence when releases were conducted at a 1:1 (*r* = 1) release ratio for *T* = 100 days with male-only (A), bi-sex (B), and female-only (C) releases. **Figure S12.** The effect of fitness cost on FK, AP, and R&R strategies. **Figure S13.** The effects of adult mortality. **Figure S14.** The effects of larval emergence.
