# Supplementary material for: Antipathogen genes and the replacement of disease-vectoring mosquito populations: a model-based evaluation
Source: Evol Appl. 2014 Oct 10;7(10):1238–51. doi: 10.1111/eva.12219 (PMC4275095; doi:10.1111/eva.12219)
Supplement: Supplementary file 1 [file eva0007-1238-sd1.pdf]

Anti-pathogen genes and the replacement of disease-vectoring mosquito populations: a model-based evaluation of hybrid strategies

Michael A. Robert, Kenichi Okamoto, Fred Gould, Alun L. Lloyd

Supplementary Material

Stochastic Model

In the main text, we utilize an ordinary differential equation (ODE) model to explore the impacts of different transgenic strategies on competent vectors in an *Aedes aeypgti* population. Because this model is fully deterministic, any impacts of demographic stochasticity and genetic drift on the ability of strategies to reduce competent vectors long-term is ignored. To assess these potential impacts, we developed a stochastic event-driven model analogous to the ODE model. The model is defined by a number of possible events, each of which has a rate defined by terms in the ODE Model. Table S1 lists the events and corresponding rates.

Simulations of the stochastic model were conducted using Gillespie's algorithm. Each transition rate determines the probability that an event occurs in the time interval $[t, t + dt]$. Once releases of transgenic mosquitoes begin, we assume that a release event happens once per week in which $\lceil rM_9^* \rceil$ adults are added to the population, where these terms are as defined in the main text and $\lceil x \rceil$ is the ceiling function which rounds x up to the nearest individual in order to maintain discrete population numbers. The genotype to which the released adults are added depends upon the particular release strategy being implemented, and the proportion of the total released individuals assigned to each sex depends upon the sex ratio of releases being considered.

Event for genotype i	Change in State	Transition Rate
Birth of juvenile	$J_i \longrightarrow J_i + 1$	B_i
Density-independent death of juvenile	$J_i \longrightarrow J_i - 1$	$\mu_J J_i$
Density-dependent death of juvenile	$J_i \longrightarrow J_i - 1$	$J_i \left(\alpha \sum_g J_g\right)^{\beta-1}$
Emergence of adult male	$(J_i, M_i) \longrightarrow (J_i - 1, M_i + 1)$	$0.5\nu J_i$
Emergence of adult female	$(J_i, F_i) \longrightarrow (J_i - 1, F_i + 1)$	$0.5\nu J_i$ *
Death of adult male	$M_i \longrightarrow M_i - 1$	$\mu_M M_i$
Death of adult female	$F_i \longrightarrow F_i - 1$	$\mu_F F_i$

Table S1: Transition rates between compartments of the stochastic model. (* As in the ODE model, non-viable adult females are assumed to die upon emergence. See Table 1 of the main text.)

The populations we consider for the main text and the stochastic simulations are rather large. The default parameters listed in Table 2 of the main text give rise to an initial wild-type population density of $J_9^* = 10119$ juveniles, $F_9^* = 7083$ adult females, and $M_9^* = 2530$ adult males. For the derivation of these values, we refer the reader to the supplementary information of Robert et al. (2013). Note that these values will change with changes in mortality rates, the larval emergence rate, the adult emergence rate, and density-dependent parameters.

We conducted stochastic simulations for each scenario considered in the main text. For each scenario, we ran $n = 50$ realizations. In order to focus on scenarios in which total population extinction did not occur when strategies involving population reduction were implemented, we conducted enough realizations to obtain $n = 50$ instances in which the population did not go extinct. For these cases, we report the percentage of total runs that went extinct. Unless noted, population extinction did not occur. Note that although our model predicts that population extinction may occur for some scenarios, this occurs in a closed, well-mixed, spatially homogenous population. It is likely that this model overestimates the

potential for extinction, and in fact it has previously been shown that the likelihood of population extinction is very low in a spatially heterogeneous environment, particularly in the presence of immigration (Legros et al. 2012, Okamoto et al. 2013).

Stochastic Model Results

In each of the figures below, we again observe the density of competent vectors relative to the initial frequency of competent vectors, and we present these values on a log scale. Because competent vector extinction is possible, we present the relative density of competent vectors as

$$\log_{10}\left(\frac{F_9(t)+1}{F_9^*(t)+1}\right).$$

In each figure we present the 5th percentile (lower dotted line), median (solid line), and 95th percentile (upper dotted line) for each result.

Switch time for combination strategies

As with the deterministic model, we found that the FK and FK/R&R releases were the least effective at reducing competent vector densities. R&R releases were more effective than FK/AP releases when the switch time was later. For the later switch time, AP-only releases were still the most effective followed by the R&R/AP releases (figure S1).

When the switch time was early, the stochastic model revealed that the order of efficacy of the FK/AP, R&R/AP, and AP-only strategies was not clear. While the medians were still ordered as we found in the deterministic model, when we compared the range between the 5th and 95th percentiles, the three strategies had very similar impacts on the competent vector population. This was due to the early switch time, which left a long period of AP-only releases.

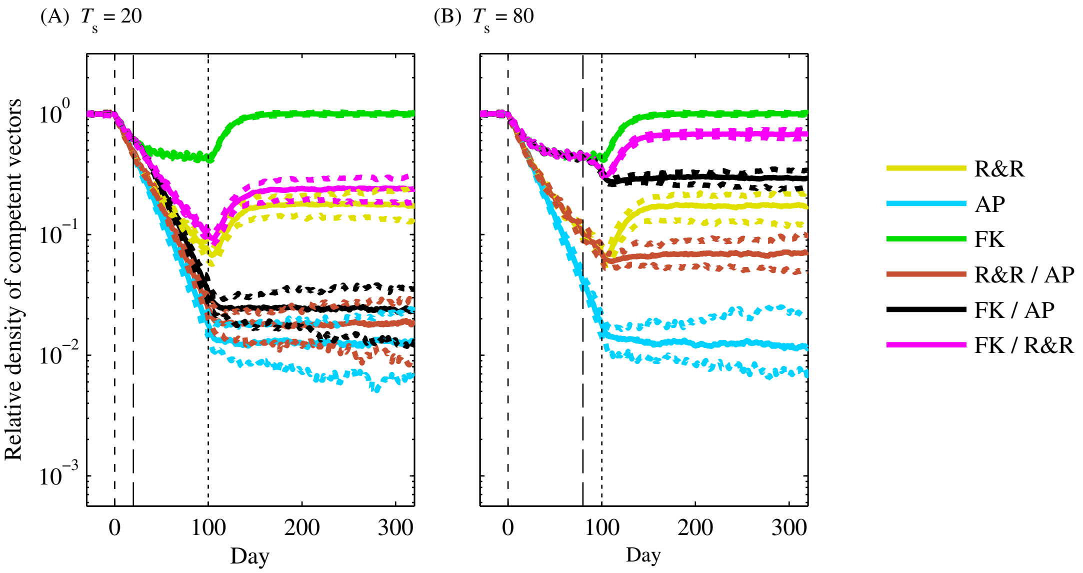

Figure S1: Relative density of competent vectors when male-only releases were conducted at a 1:1 release ratio ($r = 1$) for $T = 100$ days. Vertical black dotted lines represent the time at which releases began ($t = 0$) and ended ($t = 100$). For combination strategies, the vertical black dashed line represents the time at which a switch was made between approaches. Here, the time of switch was $T_s = 20$ (A) and $T_s = 80$ (B). All other parameter values are the default values listed in Table 2 of the main text. Solid lines represent the median of $n = 50$ realizations from the stochastic model, and dotted lines represent the 5th and 95th percentiles. Note the vertical axis is on a log scale.

Male-only, bi-sex, and female-only releases

For bi-sex and female-only releases, as well as male-only releases with a switch time of $T_s = 50$ days following the initial release, the stochastic model illustrated that R&R/AP and AP-only releases had a relatively similar effect on the long-term competent vector density, but these two strategies were more effective than the other strategies we studied (figure S2). The results from the stochastic model emphasized that R&R releases often led to population extinction when females were included in releases. In the simulations, female-only and bi-sex releases led to extinction of the population in 92.7% and 52.7% of realizations, respectively. Realizations in which total population extinction was observed have been removed from the data presented in figure S2 because we are most interested in studying the impacts of different strategies when population extinction does not occur.

One may note the solid dashed golden line that is straight at approximately 10^{-4} in Figure

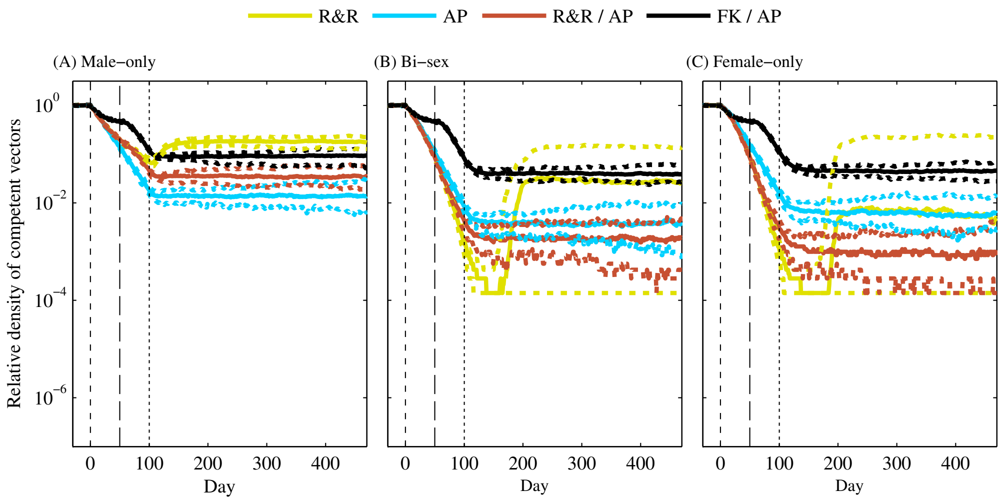

Figure S2: Relative density of competent vectors when releases were conducted at a 1:1 release ratio ($r = 1$) for $T = 100$ days with male-only (A), bi-sex (B), and female-only (C) releases. Vertical black dotted lines represent the time at which releases began ($t = 0$) and ended ($t = 100$), and the vertical black dashed line represents the time at which the switch between approaches in combination strategies occurred ($T_s = 50$). All other parameter values are the default values listed in Table 2 of the main text. Solid lines represent the median of $n = 50$ realizations (in which total population extinction did not occur) from the stochastic model, and dotted lines represent the 5th and 95th percentiles. Note the vertical axis is on a log scale.

S2B,C. This indicates that elimination of competent vectors occurred often when bi-sex and female-only R&R releases were conducted.We note, however, that on average R&R releases still led to less reduction in competent vectors than other approaches (the solid golden line in Figure S2B,C).

Impacts of density dependence

For lower values of β, the FK/AP releases clearly had less impact on long-term competent vector density that R&R/AP and AP-only releases, regardless of the release type (figure S3). When the strength of density dependence was higher, R&R/AP and AP-only releases had a similar impact on competent vector reduction for male-only releases. For bi-sex and female-only releases, R&R and R&R/AP releases generally led to greater reduction in competent vectors than AP-only releases across all except very high strengths of density dependence, whereas the differences between AP-only and R&R/AP strategies for male-only releases

were minor across all strengths of density dependence. Extinction of the population was very high for R&R bi-sex and female-only releases, particularly for populations regulated by weak density dependence (table S2).

	Extinction percentage (%)	
β	Bi-sex releases	Female-only releases
1.5	96.6	96.9
1.75	95.5	95.8
2	91.9	94.6
2.25	85.2	93.9
2.5	82.3	92.1
2.75	76.4	92.5
3	64.0	91.7
3 .25	54.7	91.3
3.5	52.9	92.8
3.75	32.0	92.4
4	40.7	91.4

Table S2: Table of percentages of population extinctions observed for R&R releases for different strengths of density dependence, β.

Sex ratio of releases

In general, the stochastic and deterministic models agreed when comparing the impacts of sex ratio of releases on long term reduction in competent vector density (figure S4). However, for higher values of β, there was not much difference between the impacts of R&R/AP and AP-only releases, especially when higher fractions of males were included in releases. R&R/AP releases often led to competent vector extinction when more females were included than males, although less competent vector extinction was observed in female-only releases than when the the male-to-female ratio was 0.25. AP-only releases, however, led to competent vector extinction more often for bi-sex releases than for other intermediate male-to-female ratios. Total population extinction was not observed for any of the realizations for this portion of the study.

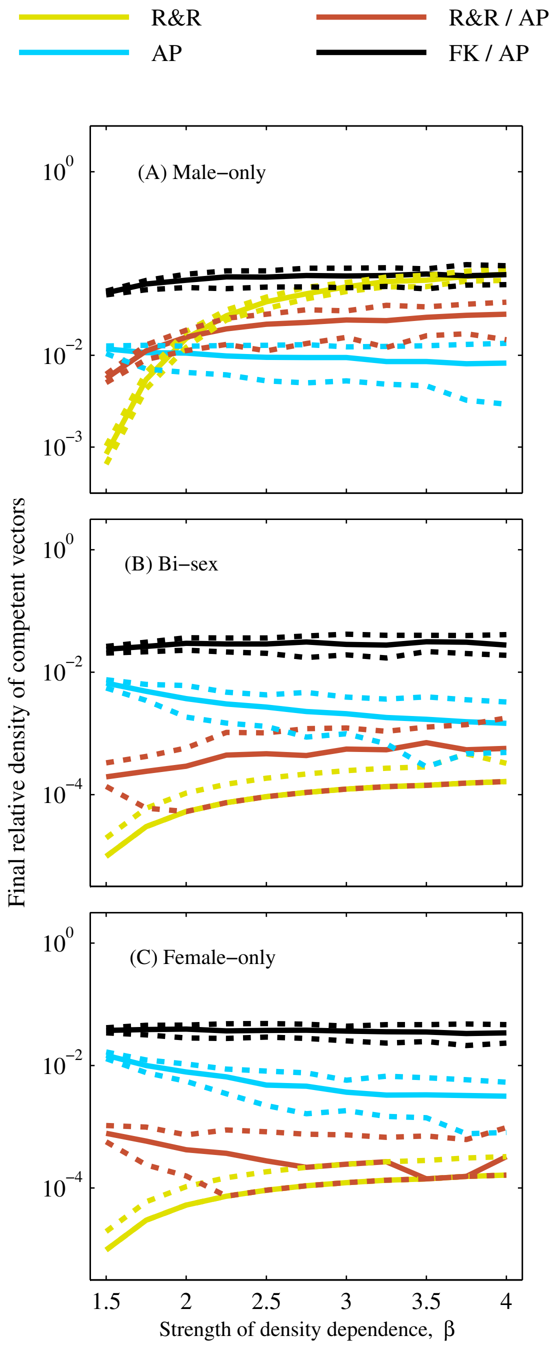

Figure S3: Final relative density of competent vectors for populations regulated by different strengths of density dependence when releases were conducted at a 1:1 ($r = 1$) release ratio for $T = 100$ days with male-only (A), bi-sex (B), and female-only (C) releases. For combination strategies, the switch occurred after $T_s = 50$ days. All other parameter values are the default values listed in Table 2 of the main text. Solid lines represent the median of $n = 50$ realizations (in which total population extinction did not occur) from the stochastic model, and dotted lines represent the 5th and 95th percentiles. Note the vertical axis is on a log scale.

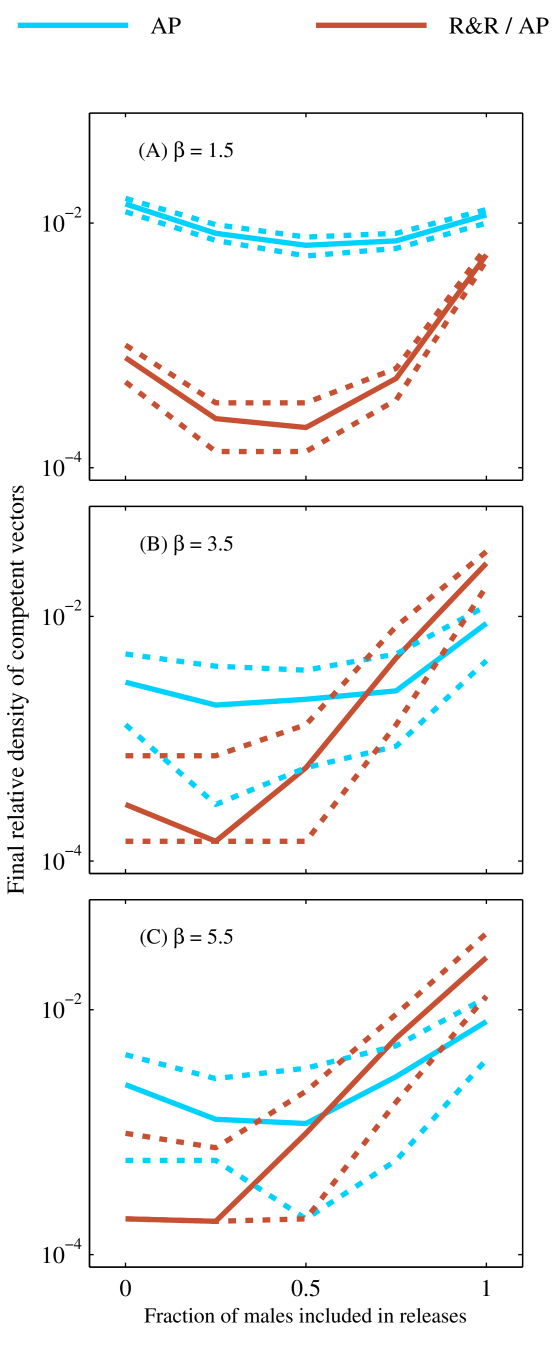

Figure S4: Final relative density of competent vectors when the sex ratio varies for releases of transgenic insects when releases were conducted at a 1:1 ($r = 1$) release ratio for $T = 100$ days. Releases were conducted into populations with a strength of density dependence of $\beta = 1.5$ (A), $\beta = 3.5$ (B), and $\beta = 5.5$ (C). For combination strategies, the switch occurs after $T_s = 50$ days. All other parameter values are the default values listed in table 2 of the main text. Solid lines represent the median of $n = 50$ realizations (in which total population extinction did not occur) from the stochastic model, and dotted lines represent the 5th and 95th percentiles. Note the vertical axis is on a log scale.

Impacts of fitness cost

We found that R&R/AP and AP-only releases had relatively the same impact on average competent vector density when the fitness cost was low (when we disregard instances in which population extinction occurred during some bi-sex and female-only releases). For bi-sex and female-only releases, R&R also had an impact on average competent vector density similar to that of R&R/AP and AP-only releases regardless of fitness cost. For higher fitness costs, all four strategies had approximately the same impact on average competent vector density in the year following releases (figure S5). Table S3 shows the percentages of extinctions observed for each fitness cost when female-only and bi-sex releases were conducted.

	Extinction percentage (%)	
c_A	Bi-sex releases	Female-only releases
0	54.5	92.2
0.1	33.6	90.6
0.2	19.0	81.0
0.3	0	51.7
0.4	0	13.0

Table S3: Table of percentages of extinctions observed during R&R releases for different values of fitness cost associated with the AP gene.

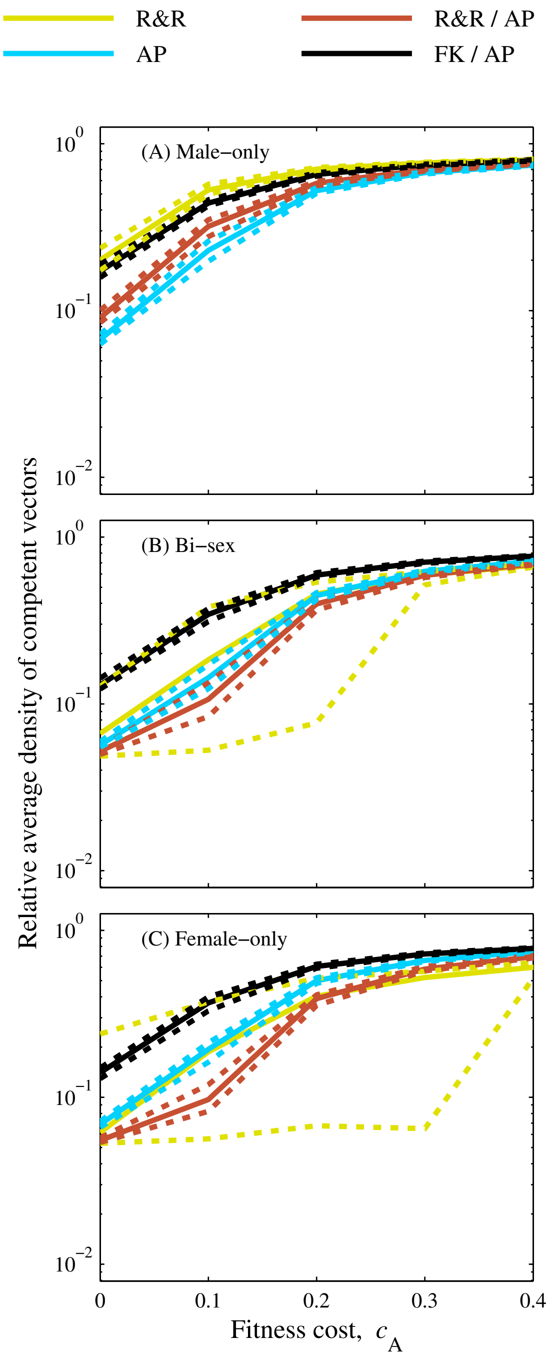

Figure S5: Relative average density of competent vectors between the time releases began ($t_0 = 0$) and one year after the final day of releases ($t_f = 465$) when the anti-pathogen gene carries a fitness cost. Releases were conducted at a 1:1 ($r = 1$) release ratio for $T = 100$ days with male-only (A), bi-sex (B), and female-only (C) releases. For combination strategies, the switch between approaches occurred after $T_s = 50$ days. All other parameter values are the default values listed in Table 2 of the main text. Solid lines represent the median of $n = 50$ realizations (in which total population extinction did not occur) from the stochastic model, and dotted lines represent the 5th and 95th percentiles. Note the vertical axis is on a log scale.

Maintenance Releases

The results of the stochastic model did not differ significantly from those of the deterministic model (figure S6).

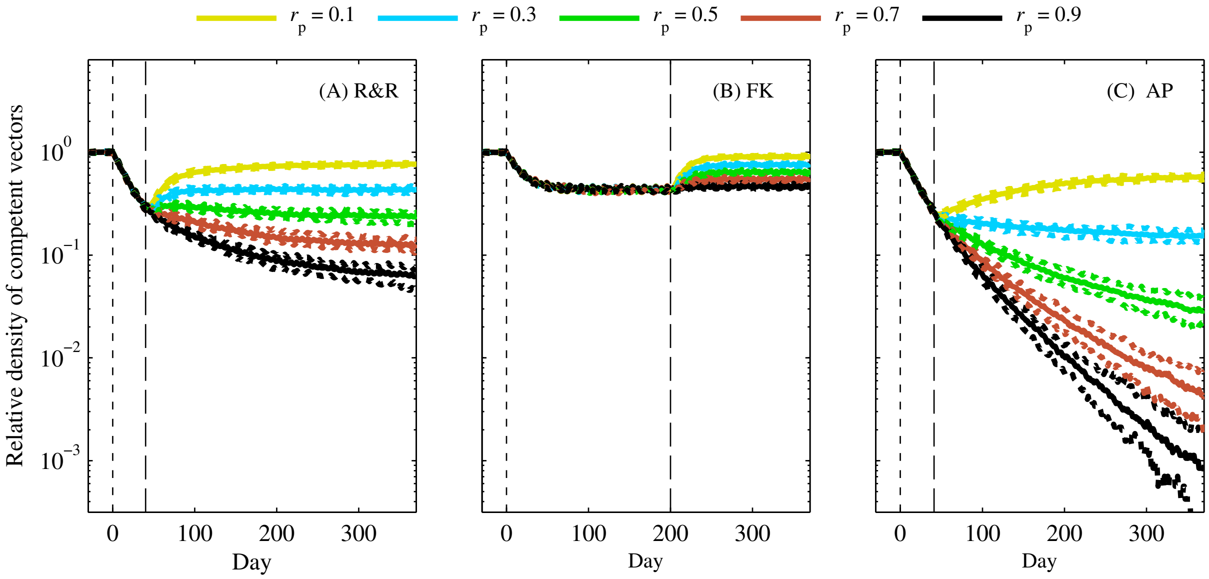

Figure S6: Relative competent vector density in populations where maintenance releases occur when the anti-pathogen gene has a fitness cost. Male-only R&R (A), FK (B), and AP (C) releases were conducted at a 1:1 ($r = 1$) release ratio until each population under control by the three different strategies reached the same density. The first dotted line represents the beginning of the original releases, and the dashed line represents the end of the original releases and the beginning of maintenance releases. Here, r_p is the fraction of the original releases ratio utilized in maintenance releases. That is, $r_p = 1$ represents continued releases at the same intensity as the original releases. $c_A = 0.2$, and all other parameter values are the default values listed in Table 2 of the main text. Solid lines represent the median of $n = 50$ realizations from the stochastic model, and dotted lines represent the 5th and 95th percentiles. Note the vertical axis is on a log scale.

Further exploration of model parameters

Release ratio, release duration, and switch time

The results presented in the main text were chosen to concisely compare the impacts of the different transgenic strategies in a number of different scenarios. Choice of release ratio, release duration, and switch time between release types can change the ranking of the strategies from least to most effective. Further, the strength of density dependence utilized as the default for this study was chosen to remain consistent with the previous work introducing and investigating the R&R strategy (Robert et al. 2013). In this supplementary material, we illustrate the influence of some of the parameters on results.

In the main text, we simulated releases for 100 days at release ratio of $r = 1$. This is primarily because some releases that include females at higher release ratios or longer durations can lead to very low densities of vectors (see Figure S3 in (Robert et al. 2013)). Because the goal of this paper is to examine strategies that can be used to reduce competent vectors when the population cannot be driven to extinction, we wish to avoid scenarios in which the total vector density is near extinction. In figure S7, we examine scenarios similar to those presented in figure 1 of the main text. Here, we simulated male-only releases at three different release ratios ($r = 1$, $r = 3$, and $r = 5$) for a duration of $T = 100$ days. We switched combination releases after $T_s = 20$ and $T_s = 80$ days (figure S7). As we observed in figure 1 of the main text, when $r = 1$, increasing the switch time from $T_s = 20$ to $T_s = 80$ changed the ordering of long-term efficacy of the R&R and FK/AP approaches. For higher release ratios, however, this change was not observed.

We studied the effects of release ratio and release duration on each of the six strategies by varying the release ratio and release duration while holding the total release number constant (figure S8). For combination approaches, we considered switch times of 1/4 ($T_s = 0.25T$) and 3/4 ($T_s = 0.75T$) of the total release duration. We considered male-only, bi-sex, and female-only releases. For all release types, longer releases of AP-only individuals led to greater long-

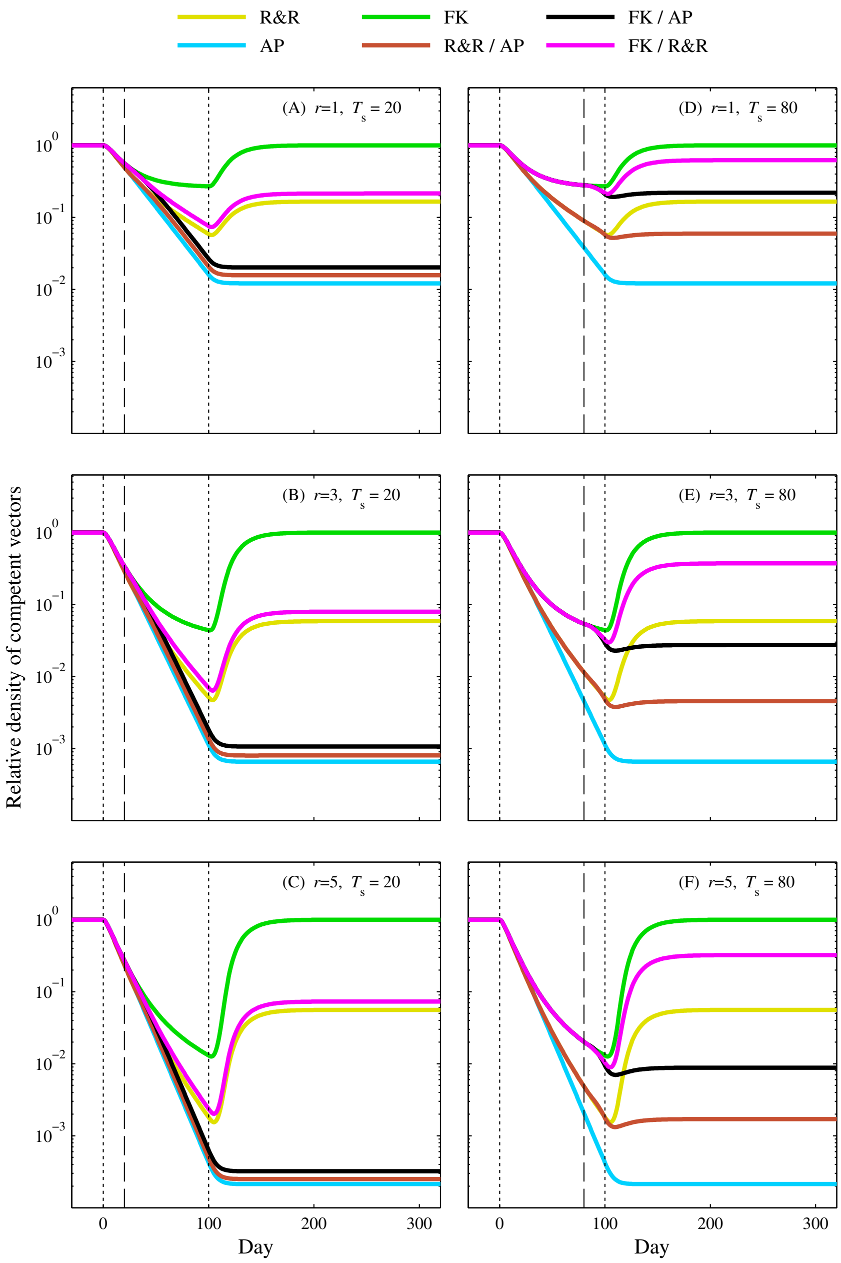

Figure S7: The effects of release ratio and switch time on male-only releases. Releases were conducted for a total of $T = 100$ days at three different release ratios: $r = 1$ (A,D), $r = 3$ (B,E), and $r = 5$ (C,F). Here the switch times are $T_s = 20$(A,B,C) and $T_s = 80$(D,E,F) days. All other parameter values are the default values listed in Table 2 of the main text. Note the vertical axes are on a log scale.

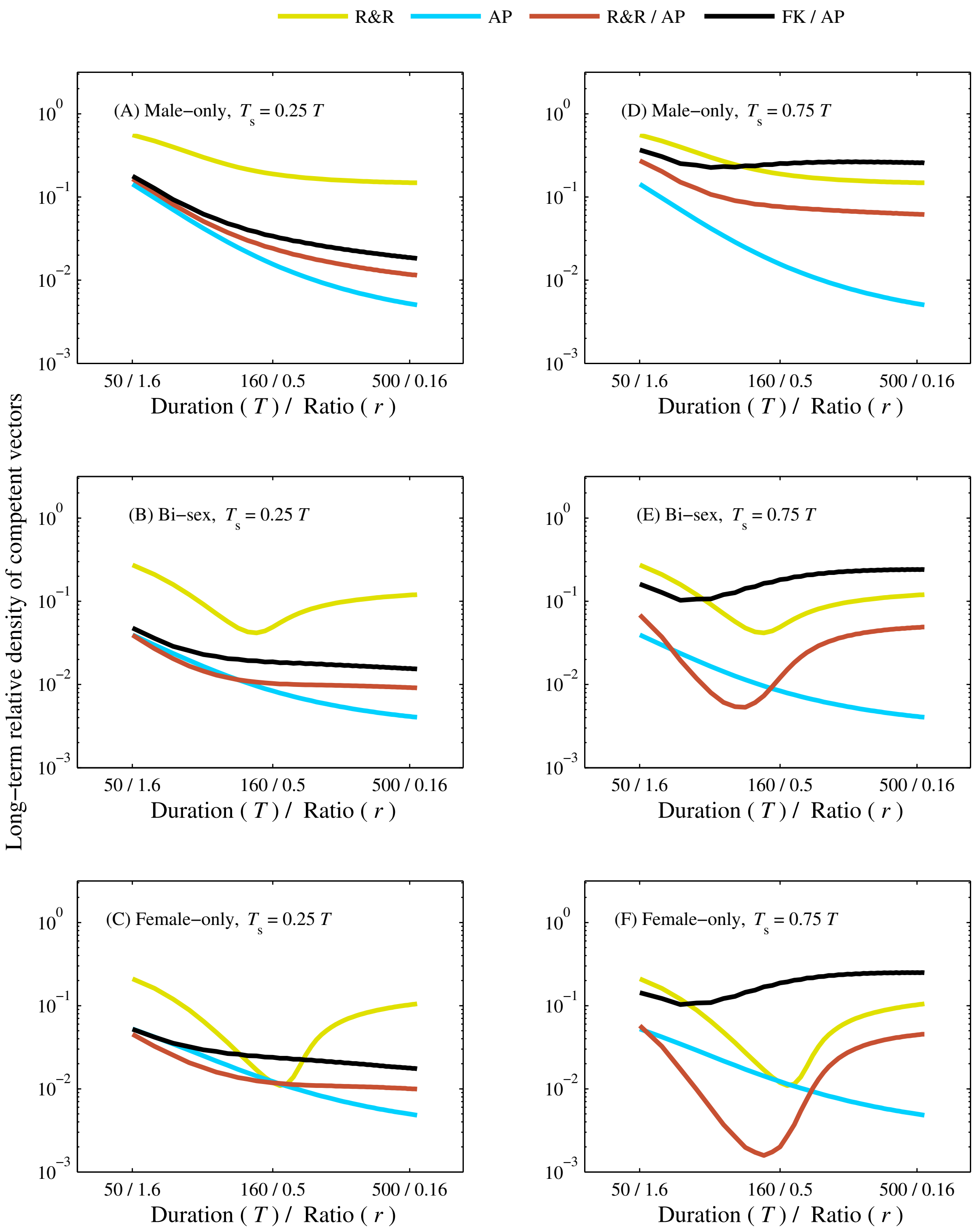

Figure S8: Long-term relative competent vector population density resulting from release scenarios that arise from different combinations of release ratio and release duration with male-only (A,D), bi-sex (B,E), and female-only releases (C,F). For combination strategies, the switch occurs after 1/4 of the total release duration (A-C) or 3/4 of the total release duration (D-F). All other parameter values are the default values listed in Table 2 of the main text. Note the vertical axes are on a log scale.

term reduction than shorter AP-only releases, and among all of the release strategies, AP-only releases led to the greatest long-term reduction in competent vectors when the release duration was longer, whereas R&R/AP releases led to the greatest long-term reduction for short, intense releases when females were included in releases. For male-only releases, AP-only and R&R-only strategies led to more reduction as the release duration increased, as did R&R/AP approaches, regardless of the switch time for combination strategies. For early switch times, FK/AP strategies led to more reduction as the release duration increased, but as the switch time increased, the most reduction caused by the FK/AP strategy resulted from an intermediate combination of a shorter release duration and larger release ratio. For longer switch times, there was a release ratio/duration combination for which R&R and FK/AP releases were equally effective, and as the release duration increased, R&R releases became more effective than FK/AP releases.

In bi-sex and female-only releases, FK/AP dynamics were similar to those of male-only releases, with greater reductions at larger release ratios when the switch time was early and an intermediate optimum for later switch times (figures S8, S9). For early and intermediate switch times, there were two release ratio and duration combinations for which R&R and FK/AP were equally effective for female-only releases (figure S8). Bi-sex releases exhibited similar behavior for intermediate switch times, but FK/AP was always more effective than R&R for early switch times. For both female-only and bi-sex releases with later switch times, FK/AP releases were less effective than R&R as the release duration increased and were as effective as R&R releases for one release ratio and duration combination. As discussed in Robert et al. (2013), R&R releases involving females had the most impact at intermediate combinations of release ratio and release duration. This intermediate optimum was reflected in releases involving R&R: R&R/AP releases also had the most impact on competent vector density for intermediate combinations of release ratio and release duration when switch times were longer.

For releases including females, there was at least one release ratio and duration combi-

nation for which R&R/AP and AP-only releases had a similar impact on competent vectors, regardless of the switch time. For all switch times, bi-sex releases of R&R/AP and AP-only had a similar impact for two combinations of release duration and ratio. For early and later switch times, female-only releases of R&R/AP and AP-only individuals had similar impacts for two different combinations of release duration and ratio. For female-only releases, there also were two combinations of release ratio and release duration for which R&R and AP-only strategies had similar impacts on competent vector density, with a small range of combinations for which R&R strategies led to more reduction than AP-only strategies.

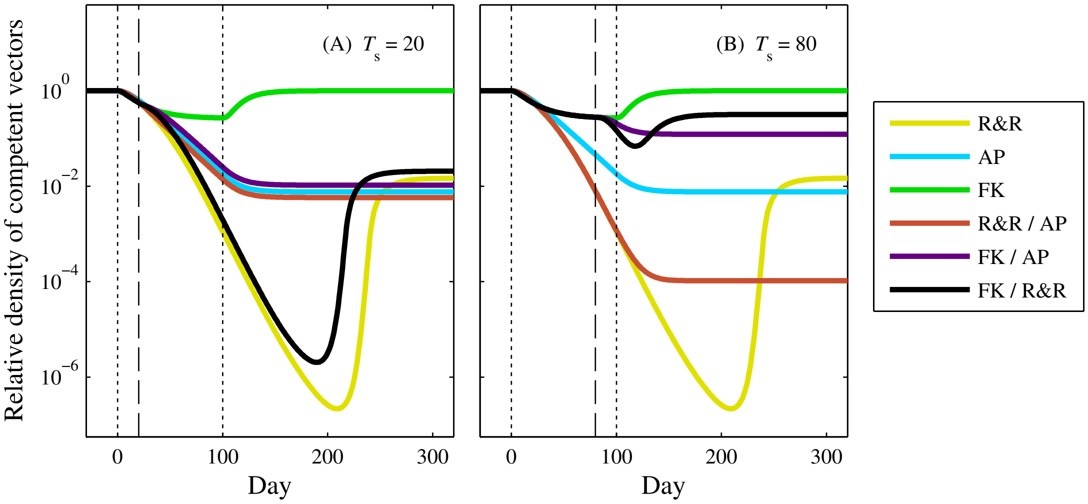

Figure S9: The effect of switch time on female-only releases. Releases were conducted for a total of $T = 100$ days at a 1:1 release ratio ($r = 1$). Here the switch times are $T_s = 20$(A) and $T_s = 80$(B) days. All other parameter values are the default values listed in Table 2 of the main text. Note the vertical axes are on a log scale.

Sex ratio, density dependence, and fitness cost

In Figure 4 of the main text, we compared the impacts of the male-to-female sex ratio on R&R/AP and AP-only releases. This specific comparison was made because these two release strategies were typically the most effective at reducing the density of competent vectors for a variety of scenarios. Here, we briefly consider the impacts of density dependence, fitness cost, and sex ratio on the efficacy of the four main release types discussed in this study (figure S10). Because we are including the impacts of fitness cost in this comparison, we measure the order of efficacy of releases by studying the impact on average relative density of competent vectors as defined in the main text.

We found that for higher strengths of density dependence and a lower fitness cost, the order of efficacy of R&R, R&R/AP, and AP strategies varied little with sex ratios in which more females were included than males (figure S10 B,C). For higher male-to-female sex ratios, AP-only typically led to the greatest reduction, followed by R&R/AP. For weak density dependence and no fitness cost, AP-only strategies actually had the least impact on average competent vector reduction for most sex ratios with the exception of very high male:female ratios (figure S10 A).

When a fitness cost was associated with the AP gene, the differences across sex ratios and strengths of density dependence was far more apparent (figure S10D,E,F). In general, R&R/AP and AP-only releases were still the most effective across all sex ratios. R&R releases were slightly more effective than AP-only releases at very high female:male ratios when density dependence was stronger (figure S10E,F). For weak density dependence, R&R and R&R/AP releases were the most effective for most sex ratios except for very high male:female ratios (figure S10 D).

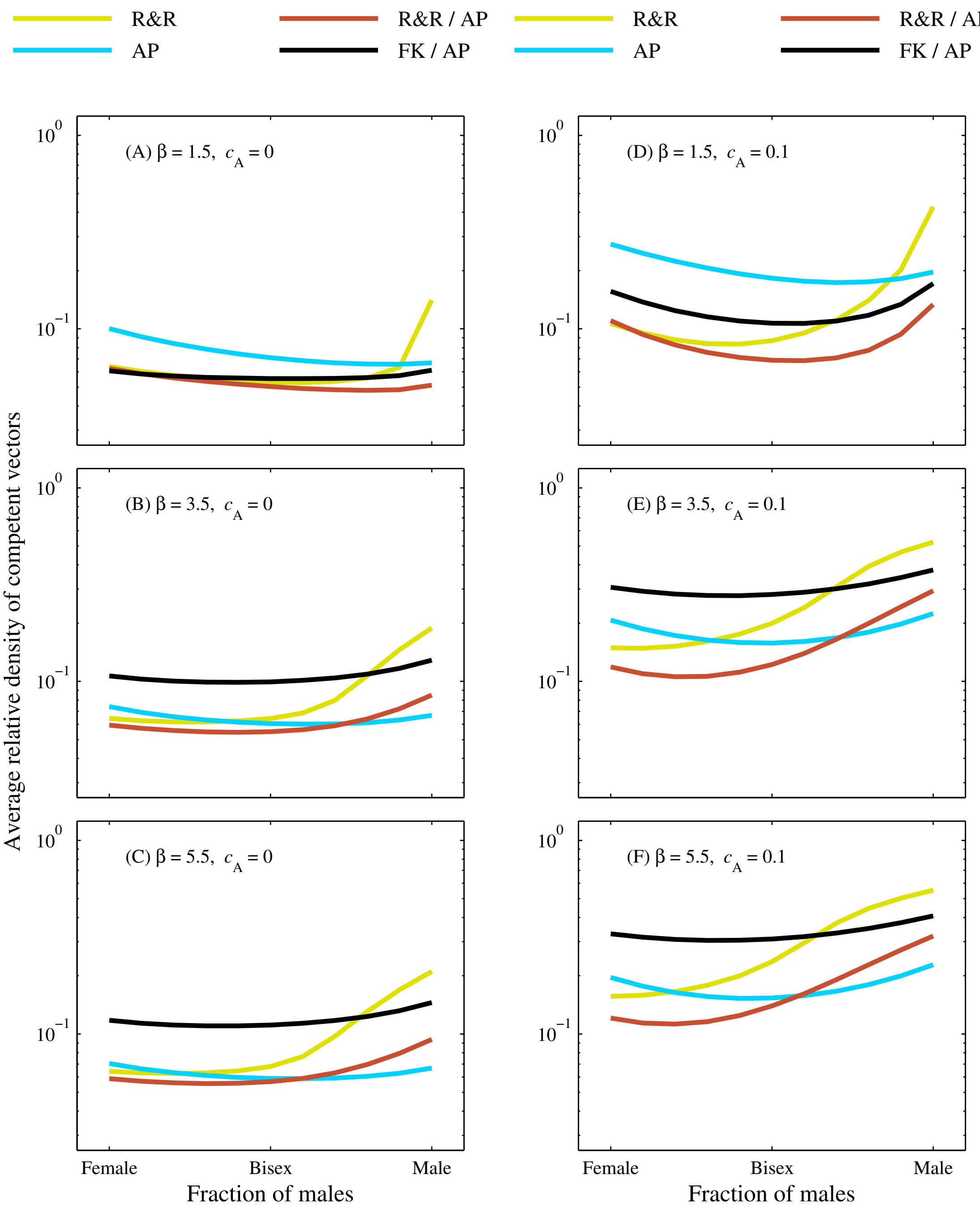

Figure S10: The effects of fitness cost, sex ratio, and density dependence on average relative density of competent vectors. The fitness cost associated with the AP gene was $c_A = 0$ (A,B,C) and $c_A = 0.1$ (D,E,F). Strengths of density dependence considered were $\beta = 1.5$ (A,D), $\beta = 3.5$ (B,E), and $\beta = 5.5$ (C,F). Releases were conducted for a total of $T = 100$ days at a 1:1 release ratio ($r = 1$). Combination strategies switched after $T_s = 50$ days. All other parameter values are the default values listed in Table 2 of the main text. Note the vertical axes are on a log scale.

Density dependence and minimum population density

Figure S11 shows the minimum relative density of competent vectors for different values of β.

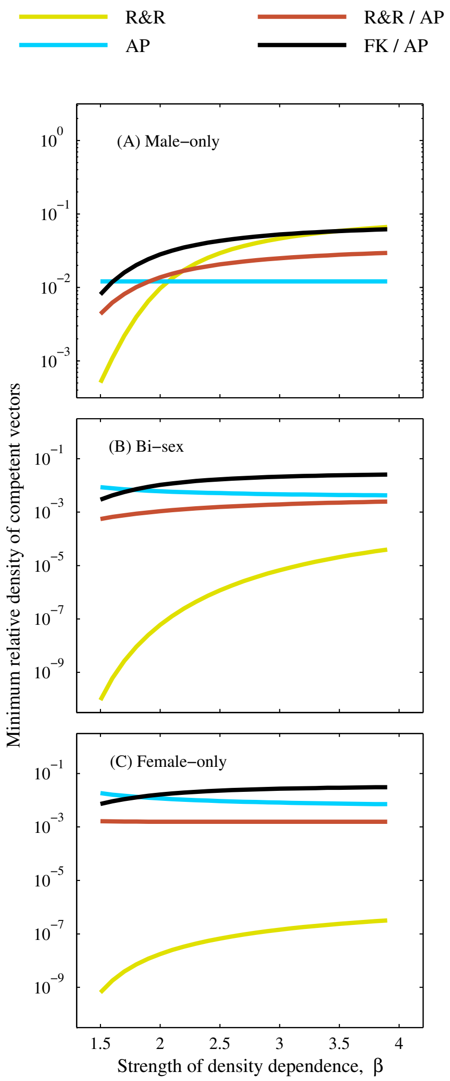

Figure S11: Minimum relative density of competent vectors for different strengths of density dependence when releases were conducted at a 1:1 ($r = 1$) release ratio for $T = 100$ days with male-only (A), bi-sex (B), and female-only (C) releases. For combination strategies, the switch occurs after $T_s = 50$ days. All other parameter values are the default values listed in Table 2 of the main text. Note the vertical axis is on a log scale.

Transient dynamics when c_A carried a fitness cost

Figure S12 shows time series dynamics for the reduction of competent vectors for male-only releases when a fitness cost is associated with the AP gene. The return to the pre-release equlibrium density following the end of releases motivates the need to compare the relative average density of competent vectors when studying the impacts of fitness cost.

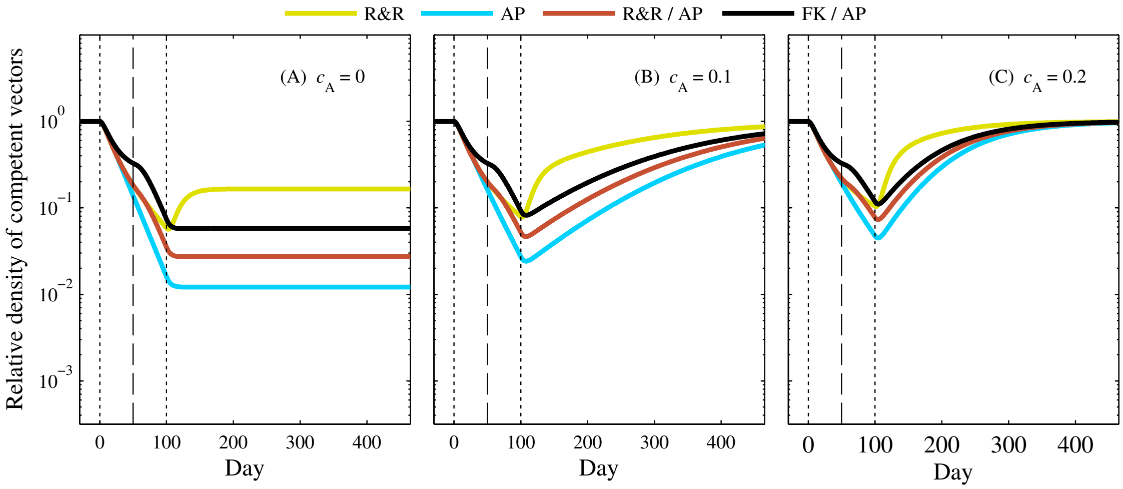

Figure S12: The effect of fitness cost on FK, AP, and R&R strategies. The fitness cost associated with the AP gene was $c_A = 0$ (A), $c_A = 0.1$ (B), and $c_A = 0.2$ (C). Male-only releases were conducted for a total of $T = 100$ days at a 1:1 release ratio ($r = 1$). Combination strategies switched after $T_s = 50$ days. All other parameter values are the default values listed in Table 2 of the main text. Note the vertical axes are on a log scale.

Impacts of adult mortality and larval emergence

Increases in adult mortality led to minor differences in the order of efficacy of the four strategies, particularly for bi-sex and female-only releases (figure S13). When adult mortality was very high, AP-only releases were the most effective at reducing competent vector density for single-sex and bi-sex releases. For intermediate values of adult mortality, female-only R&R releases were slightly more effective at reducing competent vector density than corresponding AP-only releases.

The larval emergence rate had very little impact on the order of efficacy of the four strategies (figure S14). This is likely because release ratios are determined relative to the

population size which is strongly influenced by the larval emergence rate.

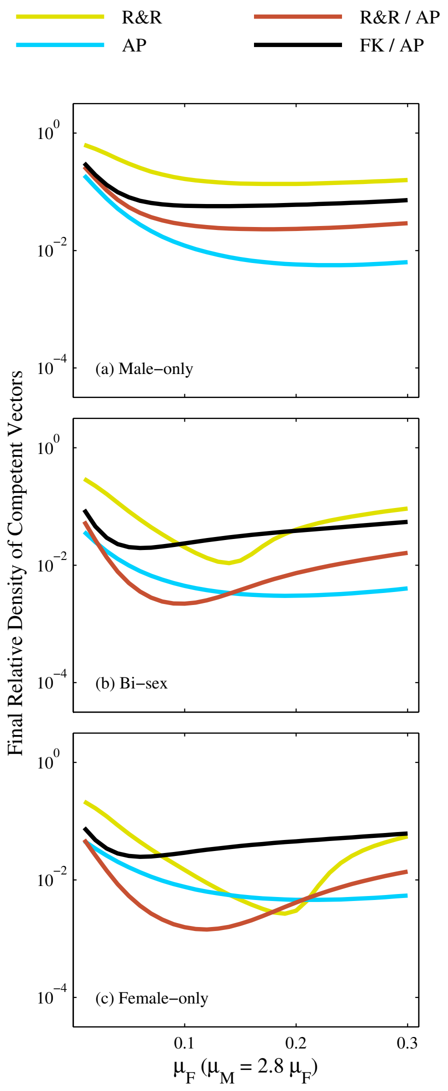

Figure S13: The effects of adult mortality. Female mortality varies along the horizontal axis and male mortality follows the relationship $\mu_M = 2.8\mu_F$ as in the main text. Male-only (A), bi-sex (B), and female-only (C) releases were conducted for a total of $T = 100$ days at a 1:1 release ratio ($r = 1$). Combination strategies switched after $T_s = 50$ days. All other parameter values are the default values listed in Table 2 of the main text. Note the vertical axes are on a log scale.

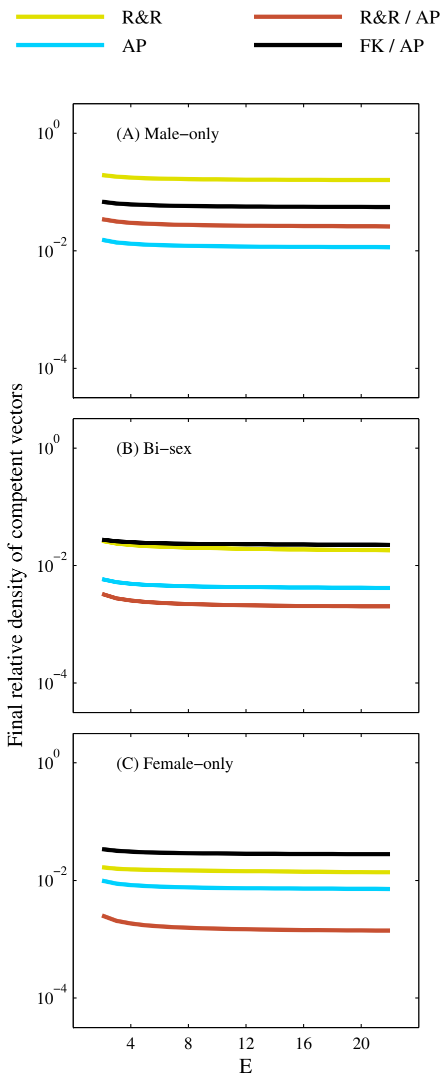

Figure S14: The effects of larval emergence. Larval emergence rate varies along the horizontal axis. Male-only (A), bi-sex (B), and female-only (C) releases were conducted for a total of $T = 100$ days at a 1:1 release ratio ($r = 1$). Combination strategies switched after $T_s = 50$ days. All other parameter values are the default values listed in Table 2 of the main text. Note the vertical axes are on a log scale.

References

Legros, M., C. Xu, K. Okamoto, T.W. Scott, A.C. Morrison, A.L. Lloyd, and F. Gould. 2012. Assessing the feasibility of controlling *Aedes aegypti* with transgenic methods: a model-based evaluation. PloS One 7 (12): e52235.doi:10.1371/journal.pone.0052235.

Okamoto, K.W., M.A. Robert, A.L. Lloyd, and F. Gould. 2013. A reduce and replace strategy for suppressing vector-borne diseases: insights from a stochastic, spatial model. PLoS ONE 8 (12): e81860. doi:10.1371/journal.pone.0081860.

Robert, M.A., K. Okamoto, A.L. Lloyd, and F. Gould. 2013. A reduce and replace strategy for suppressing vector-borne diseases: insights from a deterministic model. PloS One 8 (9): e73233. doi:10.1371/journal.pone.0073233.
